# Supplementary material for: The effects of genital myiasis on the diversity of the vaginal microbiota in female Bactrian camels
Source: BMC Vet Res. 2022 Mar 5;18:87. doi: 10.1186/s12917-022-03189-5 (PMC8897907; doi:10.1186/s12917-022-03189-5)
Supplement: Supplementary file 5 — Additional file 5. [file 12917_2022_3189_MOESM5_ESM.zip › MPL201709200_16s_yy/Treat1/css/js_css/jsPDF-master.zip!/jsPDF-master/examples/annotation/test_annotation-magFactor.pdf]

Table of Contents

Page 2
[100%] [200%] [50%] [Fit] [FitH] [FitV]
Page 3
[100%] [200%] [50%] [Fit] [FitH] [FitV]
Page 4
[100%] [200%] [50%] [Fit] [FitH] [FitV]
Page 5
[100%] [200%] [50%] [Fit] [FitH] [FitV]
Page 6
[100%] [200%] [50%] [Fit] [FitH] [FitV]
Page 7
[100%] [200%] [50%] [Fit] [FitH] [FitV]
Page 8
[100%] [200%] [50%] [Fit] [FitH] [FitV]
Page 9
[100%] [200%] [50%] [Fit] [FitH] [FitV]

Page 2

Goto First Page
Goto External URL

Page 3

Goto First Page
Goto External URL

Page 4

Goto First Page
Goto External URL

Page 5

Goto First Page
Goto External URL

Goto First Page
Goto External URL

Goto First Page
Goto External URL

Goto First Page
Goto External URL

Goto First Page
Goto External URL
